# Vitamin D Levels and Their Association With Periodontitis in Women With Polycystic Ovary Syndrome: A Cross-sectional Study

**DOI:** 10.7759/cureus.82268

**Published:** 2025-04-14

**Authors:** Durga Sahithi, Sreenivas Nagarakanti, Sravya Sri Prudhvi, Sumanth Gunupati, Sukrutha Biradavolu, Bhagyasri Chiruvella, Rishitha G, Tejasri Gudur, Charitha Neravati

**Affiliations:** 1 Department of Periodontology, Narayana Dental College and Hospital, Nellore, IND

**Keywords:** cross-sectional study, pcos, periodontitis, vitamin d, vitamin d deficiency

## Abstract

Background and aim

Remarkable evidence supports the hypothesis that vitamin D influences and prevents the sequelae of periodontal disease. Its deficiency has also been found among patients with polycystic ovary syndrome (PCOS), and it could be attributed to the polymorphisms in the vitamin D receptor (VDR) gene. Hence, the goal of this study is to assess the periodontal health of PCOS participants and investigate the serum vitamin D levels in patients with PCOS and periodontitis.

Methods and material

A cross-sectional study was conducted on 100 female participants between the ages of 18 to 40 years and were equally divided into four groups: Group 1: participants with periodontitis only; Group 2: participants with PCOS only; Group 3: participants with periodontitis and PCOS; and Group 4: participants without periodontitis and PCOS.

Results

Serum vitamin D levels in Group 1 (35.40±3.862), Group 2 (31.20±5.888), Group 3 (32.12±3.811), and Group 4 (33.24±5.885) presented no statistically significant differences among the groups. Many people in all study groups had lower levels of serum vitamin D. In PCOS individuals, there is no discernible decline in periodontal health.

Conclusions

In this study, it was concluded that serum vitamin D levels do not significantly correlate with the prevalence of PCOS or periodontitis.

## Introduction

Periodontitis is a multifaceted, intricate inflammatory condition caused by multiple factors and microorganisms. It is characterized by inflammation triggered by the host in response to bacterial imbalance [1-3]. This disease leads to the degradation of periodontal tissues in the vicinity, gradual erosion of alveolar bone and supporting ligaments, and may eventually cause tooth loss [4]. Ranking as the sixth most widespread disease worldwide, periodontitis has significant societal, economic, and systemic implications. It stands as one of the most prevalent chronic inflammatory disorders among adults [5].

A common endocrine and metabolic condition affecting women during their reproductive years is polycystic ovary syndrome (PCOS). This disorder is characterized by hyperandrogenism (HA), dysfunction in ovulation (OD), and polycystic ovarian morphology (PCOM) [4]. The incidence of PCOS among women of childbearing age ranges from 6% to 20%, depending on the criteria used for diagnosis [6].

Vitamin D is a fat-soluble nutrient that is primarily synthesized in the skin upon exposure to sunlight’s ultraviolet rays, with dietary sources accounting for only <10%-20% of the total intake [7]. Beyond its well-known role in regulating calcium balance, vitamin D has been shown to possess significant immunomodulatory properties. This is attributed to its anti-inflammatory effects, which are achieved through the suppression of cytokine production [8].

Women with PCOS are more likely to experience vitamin D deficiency (VDD). Research indicates a higher occurrence of VDD among PCOS patients [9]. Current findings suggest that VDD may play a role in causing insulin resistance (IR) and metabolic syndrome in women with PCOS. This hypothesis is reinforced by the fact that the vitamin D receptor (VDR) influences over 4% of the human genome, including genes involved in glucose metabolism [10].

Currently, to our knowledge, there are no indexed studies that investigated the interrelationship between vitamin D status in periodontitis and PCOS together. Therefore, the current study is designed to assess whether there exists a relationship between vitamin D in patients with periodontitis and PCOS.

## Materials and methods

A cross-sectional study was designed and conducted among 100 female participants, aged 18-40 years, enrolled in the Department of Periodontology (Narayana Dental College & Hospital) and Department of Obstetrics and Gynecology (Narayana Medical College & Hospital) during the period December 2021 to October 2022. The institutional ethical committee (IEC Narayan Dental College and Hospital) approved this study protocol (Approval number: IEC/NDCH/2020/P-12). Prior to the initiation of the study, all participants were explained about the study and provided their written informed consent. The study was done based on the guidelines by the Declaration of the World Medical Association 1975 in Helsinki, which were revised in 2000. The study was reviewed and verified following the STROBE (STrengthening the Reporting of OBservational Studies in Epidemiology) guidelines.

The criteria for including the participants with periodontitis (Group 1) [11,12] are: the presence of at least 20 teeth, a minimum of 30% of examined sites with a clinical attachment loss (CAL) of ≥3 mm, and probing depth of pocket (PD) ≥5 mm and presence of ≥40% sites with bleeding on probing (BOP). The criteria for including the participants with PCOS (Group 2) are: PCOS is diagnosed after taking a history regarding the clinical signs, physical examination, laboratory tests, and ultrasound observations which will be performed by a gynecologist and radiologist [13]. Participants in the healthy group (Group 4) were selected based on the following criteria: healthy women matched for age, did not use any medication that could alter periodontal status in the past 6 months, no sites with PD ≥3 mm or CAL less than 1 mm or no radiographic signs of bone loss and with no irregular menstrual cycles without clinical or biochemical presentation of HA or polycystic ovaries. Participants with periodontitis and PCOS comprise Group 3. Participants were excluded if they had used tobacco in any form, used oral contraceptives in the last 6 months, had treatment for periodontitis in the last 6 months, history of systemic disease, pregnant women, history of malignant lesions or presence of osteoporosis, use of prophylactic antibiotics for dental procedures in the previous 6 months, using drugs that could alter periodontal health in the last six months.

After the selection of participants based on inclusion and exclusion criteria, written informed consents were obtained from all the participants before the initiation of the study. One hundred participants were recruited based on inclusion and exclusion criteria and were grouped into four groups Group 1 (Test Group): Participants with periodontitis only, Group 2 (Test Group): Participants with PCOS only, Group 3 (Test Group): Participants with periodontitis and PCOS, Group 4: Healthy controls (Participants without periodontitis and PCOS).

Clinical data collection

Vitamin D intake was assessed as an international unit (IU) per day using a self-administered food frequency questionnaire (FFQ), which had previously been validated for the evaluation of vitamin D consumption in adults [14-16]. The FFQ that was used in the current study examined specifically the frequent usage of multivitamin supplements. It also included various elements which aimed at determining the typical common food intake. All patients who were enrolled had their clinical and medical features (sex, age, body mass index (BMI), and a detailed medical history) and drugs evaluated. Clinical examination of the periodontal status was carried out by the investigators who were blinded to the groups. PD, CAL, and BOP [17] are the periodontal parameters that were noted on every tooth present, except for the third molars. The biochemical parameters that were evaluated are serum vitamin D levels, complete lipid profile (including low-density lipoprotein (LDL), high-density lipoprotein HDL, total cholesterol (T Chos), and triglycerides (TGL)), total testosterone (T Test), and fasting blood sugar (FBS). All qualified study participants got venous blood samples between seven and eight AM as part of the evaluation. All patients underwent biochemical evaluations after a 10- to 12-hour overnight fast. Using the photoelectric calorimetric technique, the biochemical analyzer XL 1000 (ERBA, Mannheim, Germany) evaluated random blood sugar (RBS) and lipid profiles. The ALTA CLIAlyzer, a chemiluminescence-based immunoassay (CTK Model, manufactured by CTK Biotech, USA), was used to measure the levels of serum vitamin D and testosterone. Nanograms per milliliter (ng/mL) were used to express the amounts of vitamin D.

Sample size

Sample size calculation was done using G*Power analysis with an effect size of 0.40, α error of 0.05, and power of 90. The total sample achieved was 96 and was rounded to 100, i.e., 25 per group [18].

Statistical analysis

The descriptive construction of charts and statistical analysis was performed using the statistical software IBM SPSS Statistics version 25 (Armonk, USA). The homogeneity of variances test was tested to check whether the variances assumed were homogenous or not. Post hoc comparison for variables satisfying homogeneity of variances Tukey’s test is applied, (p<0.05 to be statistically significant). In the homogeneous subsets, the distribution of means is obtained for Post hoc Tukey comparison. Further, one-way ANOVA was carried out to find the statistical significance between the groups, at p-value< 0.05 to be statistically significant (i.e., to test the null hypothesis that no statistical significance exists between the groups).

## Results

Table 1 represents the mean and standard deviations of general parameters, which include age, showing statistically significant differences among all groups. BMI showed no significance among groups.

**Table 1:** Means and standard deviation of general parameters in different groups. One-way ANOVA, *p-value<0.05 significant BMI: body mass index

Variable	Group 1	Group 2	Group 3	Group 4	p-value
Age	35.4±3.862	31.2±5.88	32.12±3.81	34.24±8.19	0.009*
BMI	24.82±3.83	22.62±3.58	23.96±4.24	25.60±4.14	0.051 (NS)

Table 2 depicts the intergroup comparison of means and standard deviation of PD, CAL, and BOP revealing statistically significant differences among the groups with regard to all parameters.

**Table 2:** Means and standard deviation of periodontal parameters of different groups. One-way ANOVA, *p-value<0.05 significant PD: probing depth; CAL: clinical attachment level; BOP: bleeding on probing

Variable	Group 1	Group 2	Group 3	Group 4	p-value
PD	5.17±0.88	1.63±.33	5.17±.76	1.8±.61	<0.001*
CAL	5.63±.73	1.83±.42	5.74±1.07	1.78±.63	<0.001*
BOP	0.73±0.21	0.34±.17	0.77±0.32	0.40±0.50	<0.001*

Table 3 shows the means and standard deviation of biochemical parameters using a way ANOVA test, in which all parameters revealed statistically significant differences among various groups except for vitamin D.

**Table 3:** Means and standard deviation of biochemical parameters of different groups. One-way ANOVA, *p-value<0.05 significant Vit D: Vitamin D; T Test: total testosterone; T Chos: total cholesterol; TGL: triglycerides; HDL: high-density lipoprotein; LDL: low-density lipoprotein; FBS: fasting blood sugar

Variable	Group 1	Group 2	Group 3	Group 4	
Vit D	26.60±13.89	26.06±11.82	25.76±7.73	26.37±18	0.993
T Test	31.47±14.30	63.05±29.74	50.56±18.38	27.49±7.48	<0.001*
T Chos	166.84±31.7	171.35±31.71	142.12±30.88	161.8±31.49	0.01*
TGL	122.67±25.01	123.53±29.14	98.88±30.22	85.28±13.43	<0.001*
HDL	51.40±13.91	51.52±17.33	37.1±8.98	66.5±7.61	<0.001*
LDL	97.24±19.03	107.09±28.26	78.84±25.13	72.90±24.07	<0.001*
FBS	90.36±14.49	90.40±11.46	97.40±18.30	96.76±4.28	0.023*

Table 4 represents the multiple comparisons of periodontal parameters using the Post hoc Tukey test among all the groups. PD and CAL revealed statistically significant differences among Group 1 with Groups 2 and 4, Group 2 with Group 3, and Group 3 with Group 4. BOP showed a statistically significant difference among Group 1 with Group 2 and 4; Group 2 with Group 3.

**Table 4:** Multiple comparisons of periodontal parameters between the groups. Post hoc Tukey *p-value<0.05 significant PD: probing depth; CAL: clinical attachment level; BOP: bleeding on probing; NS: non-significant

Parameters	Groups		Group 1	Group 2	Group 3	Group 4
PD	Group 1	Mean diff	-	3.53	0.00	3.372
		P-value	-	<0.001*	1.000(NS)	<0.001*
	Group 2	Mean diff	-	-	-3.53	-0.162
		P-value	-	-	<0.001*	0.83(NS)
	Group 3	Mean diff	-	-	-	3.372
		P-value	-	-	-	<0.001*
	Group 4	Mean diff	-	-	-	-
		P-value	-	-	-	-
CAL	Group 1	Mean diff	-	3.80	-0.114	3.8516
		P-value	-	<0.001*	0.95(NS)	<0.001*
	Group 2	Mean diff	-	-	-3.912	0.0534
		P-value	-	-	<0.001*	0.99(NS)
	Group 3	Mean diff	-	-	-	3.9656
		P-value	-	-	-	<0.001*
	Group 4	Mean diff	-	-	-	-
		P-value	-	-	-	-
BOP	Group 1	Mean diff	-	0.393	-0.0381	0.3366
		P-value	-	<0.001*	0.977(NS)	0.003*
	Group 2	Mean diff	-	-	-0.4315	-0.0568
		P-value	-	-	<0.001*	0.929(NS)
	Group 3	Mean diff	-	-	-	0.3746
		P-value	-	-	-	0.019*
	Group 4	Mean diff	-	-	-	-
		P-value	-	-	-	-

Table 5 depicts the multiple comparisons of biochemical parameters using the post hoc Tukey test among study groups. There was no statistically significant difference between vitamin D levels and FBS levels. A statistically significant difference between Group 1 with Group 2 and 4, Group 2 with Group 3, and Group 3 with Group 4 was noted regarding T Test. TGLs and LDL revealed statistically significant differences among Group 1 with Group 3; Group 2 with Group 3. There was a statistically significant difference in HDL levels between Group 1 and Groups 3 and 4, between Group 2 and Groups 3 and 4, and between Group 3 and Group 4. Additionally, T Chos levels showed a statistically significant difference between Group 3 and Group 1.

**Table 5:** Multiple comparisons of biochemical parameters between the groups. Post hoc Tukey *P-value<0.05 significant Vit D: Vitamin D; T TEST: Total testosterone; T Chos: Total cholesterol; TGLs: Triglycerides; HDL: high-density lipoprotein; LDL: low-density lipoprotein; FBS: fasting blood sugar; NS: non-significant

Parameters	Groups		Group 1	Group 2	Group 3	Group 4
Vit D	Group 1	Mean diff	-	0.538	0.837	0.329
		p-value	-	0.99 (NS)	0.99 (NS)	1.000 (NS)
	Group 2	Mean diff	-	-	0.299	-0.209
		p-value	-	-	1.000 (NS)	1.000 (NS)
	Group 3	Mean diff	-	-	-	-0.508
		p-value	-	-	-	0.999 (NS)
	Group 4	Mean diff	-	-	-	-
		p-value	-	-	-	-
T Test	Group 1	Mean diff	-	-31.6	-19.1	3.97
		p-value	-	<0.001*	0.004*	0.88 (NS)
	Group 2	Mean diff	-	-	12.5	35.56
		p-value	-	-	0.107 (NS)	<0.001*
	Group 3	Mean diff	-	-	-	23.07
		p-value	-	-	-	<0.001*
	Group 4	Mean diff	-	-	-	-
		p-value	-	-	-	-
T Chos	Group 1	Mean diff	-	-4.5	24.7	5.05
		p-value	-	0.95 (NS)	0.03*	0.94 (NS)
	Group 2	Mean diff	-	-	29.2	9.55
		p-value	-	-	0.008*	0.70 (NS)
	Group 3	Mean diff	-	-	-	-19.6
		p-value	-	-	-	0.128 (NS)
	Group 4	Mean diff	-	-	-	-
		P-value	-	-	-	-
TGL	Group 1	Mean diff	-	-0.866	23.8	37.4
		p-value	-	0.99 (NS)	0.007*	<0.001*
	Group 2	Mean diff	-	-	24.7	38.3
		p-value	-	-	0.005*	<0.001*
	Group 3	Mean diff	-	-	-	13.6
		p-value	-	-	-	0.23 (NS)
	Group 4	Mean diff	-	-	-	-
		p-value	-	-	-	-
HDL	Group 1	Mean diff	-	-0.12	14.3	-15.1
		p-value	-	1.000 (NS)	<0.001*	<0.001*
	Group 2	Mean diff	-	-	14.4	-15.0
		p-value	-	-	<0.001*	<0.001*
	Group 3	Mean diff	-	-	-	-29.4
		p-value	-	-	-	<0.001*
	Group 4	Mean diff	-	-	-	-
		p-value	-	-	-	-
LDL	Group 1	Mean diff	-	-9.86	18.4	24.33
		p-value	-	0.48 (NS)	0.04*	0.003*
	Group 2	Mean diff	-	-	28.3	34.19
		p-value	-	-	<0.001*	<0.001*
	Group 3	Mean diff	-	-	-	5.93
		p-value	-	-	-	0.825 (NS)
	Group 4	Mean diff	-	-	-	-
		p-value	-	-	-	-
FBS	Group 1	Mean diff	-	-0.48	-7.48	-6.843
		p-value	-	0.99 (NS)	0.20 (NS)	0.273 (NS)
	Group 2	Mean diff	-	-	-7.00	-6.36
		p-value	-	-	0.246 (NS)	0.32 (NS)
	Group 3	Mean diff	-	-	-	0.64
		p-value	-	-	-	0.99 (NS)
	Group 4	Mean diff	-	-	-	-
		p-value	-	-	-	-

## Discussion

This cross-sectional study was conducted to investigate whether there exists a noteworthy relationship between serum vitamin D levels, periodontitis, and PCOS. The main objectives of this study were to assess if vitamin D levels have an impact on periodontitis and tooth loss if there exists any interplay between serum vitamin D and PCOS, and if serum vitamin D levels have an impact on periodontal status in patients having PCOS. This study also aimed to determine whether the patients who are periodontally healthy and without PCOS have a normal vitamin D level. A review re-assessing the influence of vitamin D levels on the incidence of periodontitis and tooth loss found that individuals with higher vitamin D levels had a lower incidence of periodontitis [19].

The lower levels of vitamin D had an increased incidence and increase in severity of aggressive periodontitis patients as vitamin D is an essential component for maintaining bone homeostasis [20]. Vitamin D in a dose-dependent manner had a notable reduction in inflammation of the periodontium by selectively stimulating the release of cytokines, monocytes, and T-helper lymphocytes, and enhanced production of peptides like cathelicidin, defensins from macrophages was noted to possess specific anti-inflammatory, antimicrobial activity [18,21].

Vitamin D decreases virulence and selectively inhibits a key periodontal pathogen, thereby significantly reducing the inflammatory response and periodontal damage during periodontal disease [22]. Hence, our study aimed to investigate the association between vitamin D and periodontitis.

In this cross-sectional study, we did not report a significant relation between vitamin D status and the severity of periodontitis. The results of the current study reported that the mean value and standard deviation value of serum vitamin D levels for four groups are 26.6±13.8 (Group 1), 26.06±11.8 (Group 2), 25.7±7.7 (Group 3), and 26.37±18 (Group 4). Group 3 shows low serum vitamin D levels when compared to other groups without significant variations between the groups.

The outcomes of our study also showed no significant relation between vitamin D status and the age of the participants, which contradicts the results of a previous study where they reported a correlation with the age of the patient, the severity of periodontitis, and vitamin D levels [2].

Our findings contradict the results of several observational studies that tested the association between VDD and periodontitis and PCOS [3,5,20,21]. These studies reported an increased risk for the incidence of periodontitis with VDD.

In a study, the relationship between VDD and periodontitis in Korean adults was investigated among participants aged >60 years, and it concluded that VDD was not associated with the incidence of periodontitis [23].

Furthermore, a study conducted among the Chinese population to know if an association exists between plasma calcifediol concentrations and the occurrence of aggressive periodontitis reported high serum vitamin D levels in individuals with aggressive periodontitis in comparison to healthy controls [24]. Not many studies have focused on assessing the periodontal parameters in women having PCOS and the relation between vitamin D status and PCOS.

A study conducted to know the occurrence of periodontal disease among women having PCOS when compared to healthy women showed higher BOP rates in patients with PCOS. Moreover, there was increased loss of attachment in the patients with the PCOS group in comparison to healthy controls [25]. This could be because of the increased susceptibility to the initiation of inflammatory reactions and processes, which are vital in the development and progression of periodontal disease. A cross-sectional study done to assess if VDD is associated with any metabolic risk factors among PCOS women showed that VDD is more common among women with PCOS and is increased in the presence of obesity and IR [26]. These findings do not correlate with our study outcomes.

A systematic review and meta-analysis were done to evaluate the vitamin D levels in patients with PCOS and observed that serum vitamin D levels are correlated to metabolic and hormonal disturbances in women with PCOS with significant variation in serum vitamin D levels among women with and without PCOS. However, no significant improvement was seen after supplementation with vitamin D in metabolic and hormonal functions. This study did not demonstrate the association of significantly low vitamin D levels among women with PCOS compared to non-PCOS controls [9]. Several studies reported various factors, including seasonal fluctuation of vitamin D, nutritional problems, ethnic differences, and chronic systemic problems that could be attributed to shifts in vitamin D levels.

Low levels of vitamin D have been linked to various oral diseases, including dental caries, oral lichen planus, and recurrent aphthous stomatitis. Its immunomodulatory effects enhance the body’s ability to combat harmful oral bacteria and reduce inflammation, promoting healthier oral tissues and potentially aiding in the prevention and management of these conditions.

The strength of this clinical study was the assessment of the vitamin D serum levels in correlation with the severity, distribution, risk of progression, and most crucial clinical parameters of periodontitis (PD, CAL, BOP) and biochemical parameters (Vit D, T Test, T Chos, TGLs, HDL, LDL, FBS). To our knowledge, no such detailed analysis has been performed in previous studies. Another advantage of the study is the selection of study participants, where healthy individuals were also included in the study to compare and understand the outcomes.

The limitation of the study is the size of the sample, which would have been increased. This study involved a single-time vitamin D level assessment, which does not benefit long-term analysis. Although the authors had put effort into minimizing the risk of bias, limiting a single clinical center might be disadvantageous. Further investigations with larger independent cohorts, as well as the inclusion of diverse ethnic populations, and different time intervals, are necessary to further clarify if there exists a relationship between vitamin D and periodontitis and PCOS.

## Conclusions

To date to our knowledge, there are no other indexed studies investigating the interrelationship between vitamin D status in combination with periodontitis and PCOS. This study concluded that there is no significant association between serum vitamin D levels and the prevalence of periodontitis. This further demonstrated that there is no association between serum vitamin D and PCOS. However, future studies because serum vitamin D levels change with time are needed to confirm these findings. The inclusion of various other factors, along with the serum vitamin D levels, might redefine the conclusions obtained in this study.
